# Spatiotemporal distribution and diversity of pathogenic *Vibrio* species in estuarine recreational waters of southeast Louisiana

**DOI:** 10.1128/aem.01944-25

**Published:** 2026-02-11

**Authors:** Annika Nelson, Fernanda Mac-Allister Cedraz, Katie Vigil, Joshua Alarcon, Tiong Gim Aw

**Affiliations:** 1 Department of Environmental Health Sciences, Celia Scott Weatherhead School of Public Health and Tropical Medicine, Tulane University 5783, New Orleans, Louisiana, USA; University of Delaware, Lewes, Delaware, USA

**Keywords:** *Vibrio cholerae*, *Vibrio vulnificus*, *Vibrio parahaemolyticus*, recreational water, environmental reservoir, nanopore sequencing

## Abstract

Although several studies have reported the occurrence of *Vibrio* species in the Gulf Coast of the United States (U.S.), relatively little is known about the distribution of multiple pathogenic *Vibrio* species in Lake Pontchartrain in Louisiana, the second largest estuary in the U.S. In this study, bacterial culture, quantitative PCR (qPCR), and long-read sequencing methods were used to assess the prevalence, distribution, and diversity of *Vibrio* species in this recreational estuary. Water samples (*n* = 101) were collected from nine sites over 12 months from November 2023 to November 2024. During the summer months (June, July, and August), the average *Vibrio* species concentration was 5.2 × 10^4^ CFU/L. While in the winter months (December, January, and February), the average *Vibrio* concentration was 3.2 × 10^3^ CFU/L. Likewise, the temperature differed between summer and winter, with the average water temperatures being 30.39°C and 14.45°C, respectively. Linear modeling showed water temperature and salinity were found to be significant (*P* < 0.05) predictors of *Vibrio* concentrations from both culture methods and qPCR. Precipitation was found to be significantly associated with total culturable *Vibrio* abundance. The *toxR* genes of *Vibrio cholerae*, *Vibrio vulnificus*, and *Vibrio parahaemolyticus* persisted throughout the year. Bacterial isolate sequencing revealed 41 *Vibrio* species in the estuary, with *V. cholerae, V. vulnificus,* and *V. mimicus* making up the largest proportion of the community. Genes specific to the O139 serotype of *V. cholerae* were also identified in pooled isolate samples. Additionally, the *V. vulnificus* hemolysin gene was detected in 84.2% of samples throughout the year, with higher concentrations in the summer and early fall months. Overall, diverse *Vibrio* species were detected in this recreational estuary under a broad range of environmental conditions, underscoring the value of using both culture- and molecular-based methods to surveil these pathogens of public health concern.

**IMPORTANCE:**

Globally, the diverse bacterial genus *Vibrio* is an important group of pathogens in coastal water environments. These bacteria are responsible for waterborne and seafood-borne illnesses as well as skin infections from recreational activities. Despite the rising incidence of *Vibrio* infections, routine monitoring of *Vibrio* species in the environment remains limited. This gap hinders our understanding of their distribution, especially in estuarine areas, and potential public health risks linked to recreational activities. This study provides new information on the prevalence and diversity of pathogenic *Vibrio* spp. at recreational sites along the shore of Lake Pontchartrain. The findings underscore the need for regular monitoring of *Vibrio* levels in coastal water during the recreational season for early warning and educating the public on the potential exposure risks.

## INTRODUCTION

Of the many species of *Vibrio* that inhabit water systems around the world, 12 are pathogenic to humans (1). *Vibrio* species (spp.) that are most commonly associated with human gastrointestinal illness include the three species of concern, *V. cholerae*, *V. vulnificus*, and *V. parahaemolyticus* (2, 3). *Vibrio cholerae*, specifically in the O1 or O139 serotypes, produces a toxin that causes secretion of water and electrolytes from cells, resulting in potentially-deadly, watery diarrhea (4). These bacteria have been identified as the causative agent of seven pandemics, with one persisting from the 1960s to the present (5). Cases of non-cholera *Vibrio* spp. (e.g., *V. parahaemolyticus, V. alginolyticus,* and *V. vulnificus*) infections are classified as vibriosis, but different species can cause different symptoms or severity of infection (1). *V. vulnificus*, the leading cause of seafood-associated fatality in the United States (U.S.), has an 80% rate of hospitalization and a case-fatality ratio greater than 30% due to its ability to cause skin necrosis and septicemia (6, 7). From the period of 1996 to 2010, *V. parahaemolyticus* was the most commonly reported *Vibrio* spp. infection in the U.S., but it had a case-fatality ratio of less than 1% with most patients experiencing gastrointestinal illness (7). The infectious doses of these bacteria in a healthy host can range from less than 100 cells for *V. vulnificus* to 1,000 cells for *V. parahaemolyticus*, but it is important to note that these are opportunistic pathogens that often cause severe infections in immunocompromised individuals (1, 8).

*Vibrio* spp. are found in a wide range of aquatic environments, from freshwater to marine water (9). These bacteria are known to grow best in slightly salty waters (10–25 parts per thousand or ppt), but due to the diversity of the genus, *Vibrio* have been found to grow in a wide range of salinities (1–35 ppt) (10, 11). *Vibrio cholerae* grows optimally in water with lower salinity, from 2 to 8 ppt, *V. vulnificus* can grow in water with salinity between 5 and 25 ppt, and *V. parahaemolyticus* prefers water salinity of 25 ppt (11–13). Studies have shown a significant association between temperature and *Vibrio* spp. growth (9, 14, 15). Warm bodies of water (>15°C) are suitable for *Vibrio* growth, with the three species of concern reaching ideal growth levels between 25°C and 40°C (16, 17). Due to the impact of temperature on *Vibrio* growth, most infections are detected in the summer or fall months, when sea surface temperatures are the highest (17). However, these bacteria can persist in water even in months when the temperatures drop. *Vibrio* spp. can enter a viable but non-culturable (VBNC) state in response to stressful conditions such as temperature fluctuations and other environmental factors (18, 19). Unlike dead cells, VBNC cells maintain low but detectable metabolic activity (18). This state enables *Vibrio* spp. to persist during colder months, and when conditions become favorable, they can resuscitate, regaining normal metabolic function, culturability, and virulence (18).

Because of the strong correlation between warm temperatures and *Vibrio* growth, climate change has altered both the spatial and temporal range of the species. Global sea surface temperature has increased by 0.6°C in the past 40 years, and it has been shown that enclosed bodies of water experience a higher rate of warming than the open ocean (17, 20, 21). Within the next century, models have shown *Vibrio* spp. increasing its range by over 30,000 km, and the amount of time suitable for *Vibrio* growth rising by more than a month (21). Climate change has also been associated with an increase in heat waves and severe weather events like hurricanes, which are both associated with cases of vibriosis. Cases of vibriosis were associated with floodwaters from Hurricane Katrina and Hurricane Ian, both of which impacted the Gulf of Mexico region of the U.S (17).

The Centers for Disease Control and Prevention (CDC) estimates that there are 80,000 cases of vibriosis in the U.S. each year, but many of these go unreported or misdiagnosed (20). In 2019, there were 2,685 confirmed, non-cholera vibriosis cases and 11 cholera cases in the U.S. Of these, there were 159 cases caused by *V. vulnificus*, with 52% of those cases being attributed to Gulf Coast states, including 12 cases in Louisiana (22). From January to September 2025, there were 26 reported cases of *V. vulnificus* in Louisiana, and five deaths from the bacteria. Of those reported cases, 85% of patients reported wound or seawater exposure (23). These case numbers highlight the risk of *Vibrio* exposure in Louisiana’s coastal waters.

Lake Pontchartrain is an estuarine embayment that borders the northern side of the city of New Orleans and opens into the Gulf of Mexico via Lake Borgne to the east. It is a popular site for swimming, boating, fishing, and other recreational activities. Previous studies in the Gulf of Mexico showed that *Vibrios* were present in surface waters with fluctuating concentrations based on environmental conditions (24–26). The only study of *Vibrio* in southern Lake Pontchartrain after two significant hurricanes in 2005 and 2006 showed a positive correlation of *Vibrio* concentrations with temperature and turbidity (26). Furthermore, in the 20 years since *Vibrio* spp. were last investigated in Lake Pontchartrain, intense storms and climate fluctuations have affected water systems around the world, and it is important to monitor the changing behavior of these pathogens (15). Therefore, the objectives of this study were to (i) use bacterial culture methods and quantitative PCR (qPCR) to identify the presence of *Vibrio* spp. of public health concern in a recreational estuary; (ii) determine the correlations between environmental variables (temperature, salinity, dissolved oxygen (DO), fecal indicator bacteria [FBI]) and spatiotemporal distribution of *Vibrio* spp.; and (iii) use a long-read nanopore sequencing method to identify a wide range of *Vibrio* beyond those of concern as well as more virulence markers, including genes specific to *V. cholerae* O139 strains. This is the first study to use multiple methods (bacterial culture, quantitative PCR, and long-read nanopore sequencing) to characterize environmental *Vibrio* communities in Lake Pontchartrain (26). It is increasingly important to monitor these pathogens in estuaries like Lake Pontchartrain, bear the effects of warming global temperatures and high incidence of *Vibrio* infections in Louisiana lead to public health concerns (21, 23).

## MATERIALS AND METHODS

### Site description

**Fig 1:**
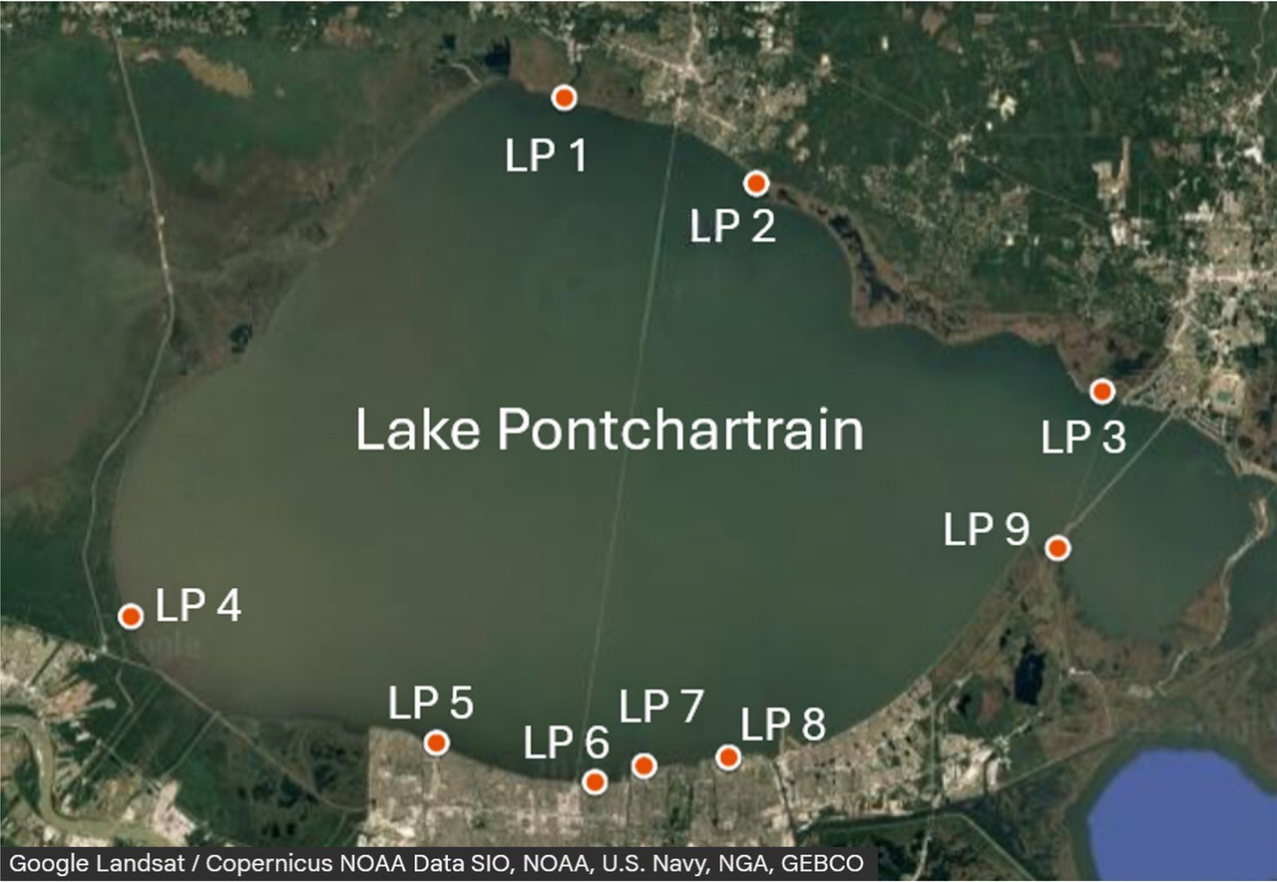
Map showing sampling sites along the shore of Lake Pontchartrain (LP). The LP 1–LP 3 are sites located on the north shore of the lake, and LP 4–LP 9 are the south shore sites.

Lake Pontchartrain is a brackish estuarine embayment located in southeast Louisiana (Fig. 1; Tables S1). It is the second largest inland estuary in the U.S., covering an area of 630 square miles (1,631 square km). The lake is an important resource, supporting agricultural and aquacultural activities, fishing tourism, and recreation (27). Water samples were collected at nine recreational sites from November 2023 to November 2024. Over the course of the study, 101 surface water samples were collected.

### Sample collection and water quality parameter measurements

During the study period, each site was sampled once a month. Two sampling trips were conducted each month, each trip collecting samples from the north shore of the lake (LP 1–LP 3) and south shore of the lake (LP 4–LP 9). Exceptions included December 2023, when no samples were collected, March 2024, when south shore samples were not collected, and November 2024, when LP 1 was not collected due to flooding (Fig. S1). At the time of sample collection, a multiparameter weatherproof meter (HANNA Instruments, Smithfield, RI) was used to measure pH, DO, salinity, and total dissolved solids (TDSs). Precipitation data were obtained from the United States Geological Survey National Water Information System at station #302415090091500 in Madisonville, Louisiana, U.S. Reported precipitation in inches (in) for the sampling date and 6 days prior were summed to provide a 7-day precipitation measurement. A 1-L grab sample of water was collected from each site using a sterile bottle (Thermo Fisher Scientific, Waltham, MA). Water samples were stored in a cooler with ice packs during transport to the lab, where they were held at 4°C until processing (<48 h). Upon return to the lab, enzyme-based methods were used to assess the presence of FIB. Briefly, the Colilert-18 reagent (IDEXX Laboratories, Westbrook, ME) was added to 100 mL of the sample in a sterile sample vessel and mixed well to dissolve. The mixture was poured into an IDEXX Quanti-tray 2000 and incubated for 18–22 h at 35°C (±0.5°C). Following incubation, wells showing yellow coloration (total coliforms) and fluorescence under 365 nm UV light (*Escherichia coli*) were counted and interpreted using the IDEXX Most Probable Number (MPN) table, with results reported as MPN/100mL in accordance with the manufacturer’s instructions. Similarly, for enumerating enterococci, 100 mL of sample was mixed with the Enterolert reagent in a sterile sample vessel (IDEXX Laboratories, Westbrook, ME), and incubated for 24–28 h at 41°C (±0.5°C). After incubation, positive wells for enterococci (fluorescent under 365 nm UV light) were counted and interpreted using the IDEXX MPN table, with results reported as MPN/100 mL in accordance with the manufacturer’s instructions.

### Enumeration of *Vibrio* spp. using culture-based assay

Within 48 h of collection, water samples were filtered for use in a bacterial culture-based assay. Water samples were diluted with 1× sterile phosphate-buffered saline (PBS) to get the sample plates within the countable range. For each sample, a 1:10 (vol/vol) and 1:50 (vol/vol) 1× PBS dilution was made to get a total volume of 100 mL for filtration. Both dilutions and 100 mL of the undiluted sample were filtered in duplicate on 0.47 mm diameter mixed cellulose ester gridded filters with 0.45 μm pore size (Pall Corporation, Port Washington, NY). For each filtration, a negative control using 100 mL of sterile 1× PBS was included. Filters were placed on thiosulfate-citrate-bile salts-sucrose (TCBS; Cat. No. 265020, BD Difco) agar and incubated at 37°C for 18–24 h. After incubation, the colonies were counted. The countable range was <200 colonies per plate, and the duplicate plates were averaged. The concentration of *Vibrio* spp. in the sample measured in colony-forming units per liter (CFU/L) was calculated by multiplying the number of colonies times the dilution factor and dividing by the volume filtered.


$$\frac{CFU}{L}=\frac{\left(number\;of\;colonies\right)\times \left(dilution\;factor\right)}{volume\;filtered\;\left(L\right)}$$

TCBS agar is traditionally employed for the enumeration of *Vibrio* spp. due to its selective properties and ability to differentiate sucrose-fermenting from non–sucrose-fermenting strains. Studies have demonstrated that this medium can also support the growth of non-*Vibrio* colonies, and culture on this medium does not account for *Vibrio* cells in a VBNC state (28–30). For this reason, samples within this study were also quantified using qPCR using the 16S rRNA gene, which is highly conserved within the genus, allowing for a higher level of specificity and for quantification of VBNC bacteria (31, 32).

### Confirmation of *Vibrio* species using polymerase chain reaction

The TCBS agar differentiates *Vibrio* spp. by sucrose fermentation and hydrogen sulfide production, resulting in distinct colony colors and morphologies. *V. cholerae* formed large yellow colonies, and *V. vulnificus* formed yellow-green colonies, whereas *V. parahaemolyticus* produced blue to green colonies. Therefore, yellow and green colonies from TCBS plates were extracted separately. Qualitative PCR assays were performed on five colonies of each color per sampling site to confirm the presence of *Vibrio* spp. PCR primers targeting the *toxR* gene of each species, which encodes a protein that regulates the expression of virulence and survival factors, including cholera toxin, outer membrane proteins, and bile resistance (33), were used (Table S2).

For DNA extraction, colonies of the same color (yellow or green) were harvested from a plate and resuspended in 100 μL of nuclease-free water in a 2 mL microcentrifuge tube. The tubes were vortexed for 5 s and then heated to 95°C in a heat block for 5 min. After heating, the tubes were centrifuged for 2 min at 12,000 rpm. The supernatant was placed in a labeled 1.5 mL microcentrifuge tube and stored at −80°C until PCR.

Positive controls for PCR were generated from the American Type Culture Collection (ATCC; Manassas, VA) stock bacteria. For the *V. cholerae* (ATCC #14033) and *V. parahaemolyticus* (ATCC # 43996) cultures, 1 mL glycerol stock was placed in 9 mL Difco Nutrient Broth (VWR, Radnor, PA) in a loosely capped 15 mL tube and incubated at 37°C for 24 h. For the *V. vulnificus* (ATCC #27562) cultures, 1 mL glycerol stock was placed in 9 mL Difco Marine Broth (VWR, Radnor, PA) in a loosely capped 15 mL tube and incubated at 37°C for 24 h. After incubation, 1 mL of broth was pelleted down via centrifugation for 1 min at 13,000 rpm. The supernatant was removed, and the pellet was resuspended in 100 mL nuclease-free water. The heat extraction method described above was used, and the DNA was stored at −80°C until PCR.

A 25 µL reaction was assembled using 12.5 µL AccuStart II PCR Supermix (Quantabio, Beverly, MA), 4 µL of forward primer (10 µM), 4 µL of reverse primer (10 µM), 2.5 mL of nuclease-free water, and 2 µL DNA template. For the non-template control, 2 µL of nuclease-free water was used instead of extracted DNA. Thermocycling conditions can be found in Table S3. Thermocycling was completed on a MyCycler Thermal Cycler (Bio-Rad Laboratories, Hercules, CA). PCR products were visualized on a 1.5% agarose gel with a SYBR Safe DNA Gel Stain (Thermo Fisher Scientific, Waltham, MA). 10 µL of PCR product was loaded with 2 µL 6× Purple Gel Loading Dye (New England BioLabs, Ipswich, MA). A 100 bp DNA ladder (Promega, Madison, WI) was loaded in the same manner and used as a standard to estimate amplicon sizes.

### Enumeration of *Vibrio* spp. using qPCR

An undiluted water sample (100 mL) was filtered through 0.4 μm pore size, 0.47 mm diameter polycarbonate filters (MilliporeSigma, Burlington, MA) for use in qPCR. DNA extraction was performed using the DNeasy PowerLyzer PowerSoil kit (QIAGEN, Hilden, Germany) according to the manufacturer’s instructions. Briefly, bacterial cells collected on the filters were resuspended and homogenized in the Mini-Beadbeater-16 (Biospec Products, Bartlesville, OK) for 45 s. Following this, the sample underwent protein precipitation, inhibitor removal, DNA binding, and wash steps. DNA was eluted in 100 μL of DNase, RNase-free water, and stored at −80°C until the qPCR assay was performed.

A SYBR Green assay with the 16S rRNA gene (Table S1) was used to determine concentrations of *Vibrio* spp. in water samples. Each 25 µL reaction consisted of 10 µL of 2 × Applied Biosystem PowerUp SYBR Green Master Mix (Thermo Fisher Scientific, Waltham, MA), 1 μL of 10 µM forward primer, 1 μL of 30 µM reverse primer, 4 μL of nuclease-free water, and 4 μL of sample or standard DNA. Thermocycling conditions are in Table S2. Thermocycling and data collection were completed on the QuantStudio 3 Real-Time PCR System (Thermo Fisher Scientific, Waltham, MA). To develop standard curves for qPCR, a gBlock gene fragment (Integrated DNA Technologies, Coralville, IA) was designed to cover the 16S rRNA region of *Photobacterium angustum*. The gBlock was diluted 10-fold using nuclease-free water with concentrations ranging from 10^7^ to 10 gene copies/5µL. Additionally, each plate included non-template controls. All samples and standards were run in triplicate on a 0.2 mL, 96-well plate. The efficiency percentage and *r*^2^ values were computed for each standard curve, with acceptable values between 90% and 110% for efficiency and ≥0.980 for *r*^2^. In this study, qPCR efficiency ranged from 93.3% to 95.9%, and *r*^2^ ranged from 0.996 to 1. Since *Vibrio* spp. contain between 9 and 14 copies of the 16S rRNA gene per cell, the number of presumed cells in each sample was calculated by dividing the gene copy number by 9.1. This number, 9.1, is the average number of 16S rRNA gene copies across *Vibrio* species (25).


$$\frac{cells}{L}=\frac{gene\;copies}{L}\times \frac{cell}{9.1\;gene\;copies}$$

qPCR was also used to quantify the *Vibrio* spp. of concern (*V. cholerae*, *V. vulnificus*, and *V. parahaemolyticus*) using primers, probes, and gblocks as listed in Table S2. For specific *Vibrio* spp. qPCR assays, 20 mL reactions consisted of 10 mL 2× PerfeCTa qPCR Toughmix Low ROX (Quantabio, Beverly, MA), 1 mL of 10 µM forward primer, 1 mL of 10 µM reverse primer, and 0.2 mL of 10 µM probe, 3.8 mL of nuclease-free water, and 4 mL of extracted DNA sample or standard DNA. Thermocycling conditions differed for each of the three species (Table S2). Thermocycling and data collection were completed on the QuantStudio 3 Real-Time PCR System (Thermo Fisher Scientific, Waltham, MA). All samples and standards were run in triplicate on a 0.2 mL, 96-well plate.

The detection rate for qPCR and PCR samples was calculated as follows:


$$Detection\;rate\;\left(\%\right)=\frac{\#\;of\;samples\;with\;gene\;detected}{total\;\#\;of\;samples\;}\times 100$$

### Genomic sequencing for culturable *Vibrio* spp. and bioinformatics

To determine the diversity of *Vibrio* spp. isolated from the estuary, bacterial colonies were harvested from TCBS Agar plates by using 1 mL of Luria-Bertani (LB) Broth (Thermo Fisher Scientific, Waltham, MA) and a sterile L-shaped loop to dislodge colonies from the filter. Broth with suspended colonies was pipetted into a 1.5 mL cryotube, and 150 μL autoclaved glycerol was added to make a 15% glycerol stock. Tubes were vortexed and stored at −80°C. This process was completed in duplicate for each sample site.

For DNA extraction, the preserved cells from each site were pooled in 2 mL tubes and spun down for 5 min at 5,000 rpm. The supernatant was discarded, and the pellet was resuspended in ZymoBIOMICS DNA/RNA Shield solution (Zymo Research, Orange, CA). DNA was extracted according to the ZymoBIOMIC DNA/RNA Miniprep Kit DNA Purification protocol. In this study, 75 μL of the ZymoBIOMICS Microbial Community Standard was also extracted using the same protocol as a positive control.

DNA samples were prepared for sequencing with the Native Barcoding Kit NBD114.24 (Oxford Nanopore Technologies, Oxford, UK). For each sequencing, non-template controls and the ZymoBIOMICS Microbial Community Standard (Zymo Research, Orange, CA) as a positive control were also included. Tapestation electrophoresis (Agilent Technologies, Santa Clara, CA) was used to get the average input fragment length (kb) in the DNA library. This fragment length was used to calculate the volume of the library that contained 50 fmol of DNA to load into a PromethION R10.4.1 Flow Cell (Oxford Nanopore Technologies, Oxford, UK) for nanopore sequencing with the MinKNOW v24.11.10 using the “fast” Dorado Basecaller Setting. Samples were sequenced for up to 72 h, the minimum quality score was set to 8, and a minimum read length was set to 1 kb.

Nanopore-generated FASTQ files were concatenated via the command line and uploaded to the IDseq mNGS Nanopore Metagenomic Pipeline v0.7. The pipeline filtered low-quality reads using fastp, removed host and human sequences with minimap2, subsampled to 1 million reads, assembled contigs using metaFlye, and aligned both contigs and unassembled reads to the NCBI nucleotide and protein databases using Minimap2 and DIAMOND (34–37). Output reports included identified taxa, total bases per taxon, percent identity, alignment length, contig counts per taxon, and average E-value. Positive control samples were reviewed to confirm the detection of organisms present in the microbial community standard. The results from the IDseq pipeline were uploaded to R (Version 2024.120 +467) for data visualization. Sequencing reads that aligned with the *V. cholerae* genome in the IDseq were also uploaded to Cholerae Finder 1.0 (https://cge.food.dtu.dk/services/CholeraeFinder/) for serotyping. Reported species were screened to have more than 1 kilobase (kb) of aligned reads and a percent identity greater than 95%, which shows high alignment with known sequences in the BLAST database.

### Statistical analysis

All statistical analyses were performed using R (Version 2024.120 +467). Data were cleaned using the dplyr and lubridate packages. The graphs were generated using ggplot2. A multiple linear regression model was used to assess the relationship between environmental factors (salinity, temperature, precipitation, DO, TDSs, and pH) and *Vibrio* spp. concentrations collected via bacterial culture (CFU/L) and qPCR (cells/L). Values for bacterial culture concentration, qPCR concentration, and pH were log-transformed for normality. The equation for the linear regressions was as follows: log (*Vibrio* concentration) = β0 + β1(Salinity) + β2(Temperature) + β3(Precipitation) + β4(Dissolved Oxygen) + β5(log(pH)) + β6(total dissolved solids) + ϵ. The package “lmtest” was used to make linear models, which were assessed for heteroscedasticity of residuals using the Breusch-Pagan test. Significance of environmental parameters on *Vibrio* spp. concentration was assessed using a type III ANOVA test where a *P*-value less than 0.05 was determined to be significant. Pairwise comparisons between enumeration and qPCR data for the months of the study and the study sites were performed using the Wilcoxon signed-rank test for non-normal data and the Bonferroni correction for significance from the package “rstatix.”

## RESULTS

### Water quality and environmental parameters

Table 1 summarizes key water quality parameters of the estuary measured during the study. Water temperature ranged from 8.27°C to 32.37°C, with seasonal variation. Median water temperature during summer months (June, July, and August) was 30.5°C, whereas during winter months (December, January, and February), median water temperature was 13.35°C (Fig. S2). Salinity ranged from 0.08 PSU to 10.01 PSU, which indicated brackish water. The DO percent saturation of water samples ranged from 51.7% to 111%. These values are based on expected solubility for a given salinity and temperature, but they can exceed 100% because of photosynthetic organisms that produce oxygen that do not immediately diffuse to reach equilibrium with the air. The highest precipitation value for the 7-day period before sample collection was 8.47 inches from Hurricane Francine in September 2024. TDSs ranged from 18 to 8,484 mg/L throughout the course of the study. Of the samples that were tested, *E. coli* was detected in all but one sample, and enterococci were detected in all samples.

**TABLE 1:** Summary of key environmental parameters of Lake Pontchartrain collected over the course of the study

Parameter	Minimum	Maximum	Median
Temperature (°C)	8.27	32.37	23.14
Salinity (PSU)	0.08	10.01	2.21
Dissolved oxygen (% saturation)	51.70	111.00	87.90
7-Day precipitation (in)	0.00	8.47	0.50
Total dissolved solids (mg/L)	18	8484	1961.5
*E. coli* (MPN/100 mL)	<1	1732.90	24.30
Enterococci (MPN/100 mL)	2	>2419.6	79.80

### Enumeration of *Vibrio* spp. in an estuarine lake

**Fig 2:**
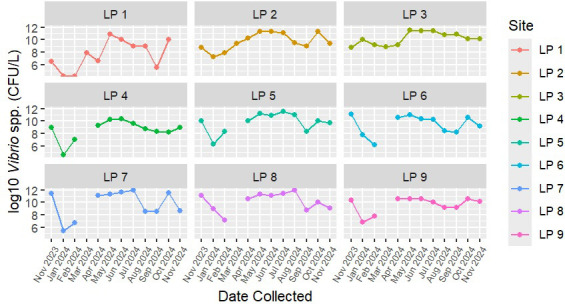
Temporal occurrence of *Vibrio* spp. in nine recreational sites as determined using culture assay with selective media. The y-axis shows the log_10_ of *Vibrio* spp. concentrations in CFU per liter. The x-axis shows sampling months, with each dot representing data from individual samples.

**Fig 3:**
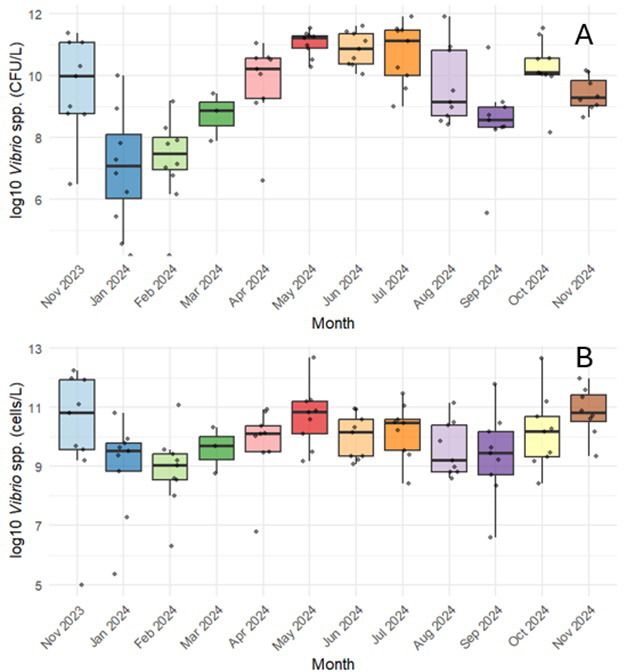
Monthly distributions of *Vibrio* spp. concentrations as determined by (**A**) bacterial culture methods in CFU/L and (**B**) qPCR in cells/L. The x-axis shows the months when the samples were collected. Each dot represents an individual sample. Box boundaries represent the lower (Q1) and upper (Q3) quartiles, and whiskers extend to the minimum and maximum non-outlier values. Temporal differences in *Vibrio* spp. concentrations were statistically significant for bacterial culture methods, with January and February showing significant differences from some spring, summer, and fall months (Table S5).

Using the culture technique, *Vibrio* spp. were detected in 98% (*n* = 99) of water samples, with bacterial counts ranging from 30 to 8.65 × 10^4^ CFU/L. Temporal variation in the prevalence of *Vibrio* spp. was observed over the course of the year (Fig. 2). Concentrations of *Vibrio* spp. were greater during the summer months (June, July, and August, *n* = 27), with the geometric mean of the concentrations being 3.2 × 10^4^ CFU/L. In the winter months (January and February, *n* = 18), the average *Vibrio* concentration was 3.1 × 10^1^ CFU/L. A two-tailed t-test showed a significant difference between these seasons (*P* = 3.7 × 10^−6^). Comparisons between individual months found that *Vibrio* spp. concentrations in January and February were significantly different from concentrations in May, June, and July (Table S5). Additionally, bacterial culture concentrations in October and November of 2024 were significantly different from February of the same year (Table S5). Quantitative PCR results showed a similar temporal pattern, with *Vibrio* DNA concentrations tending to be higher in water samples during the summer months, but none of these differences were significant with the Wilcoxon signed-rank test (Fig. 3).

**Fig 4:**
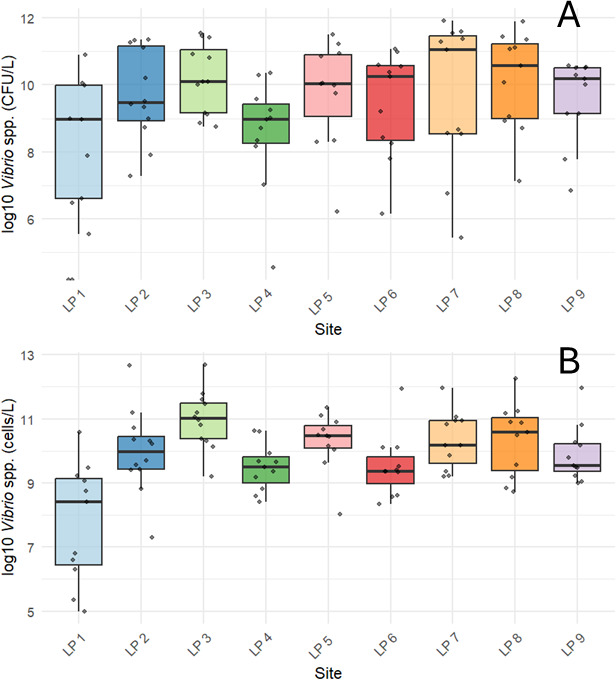
Spatial distributions of *Vibrio* spp. concentrations as determined by (**A**) bacterial culture methods in CFU/L and (**B**) qPCR in cells/L. *Vibrio* spp. concentrations varied within the north shore and south shore sites. The x-axis shows each sampling site. The LP 1–LP 3 are sites located on the north shore of the lake, and LP 4–LP 9 are the south shore sites. Each dot represents an individual sample. Box boundaries represent the lower (Q1) and upper (Q3) quartiles, and whiskers extend to the minimum and maximum non-outlier values. Pairwise comparison of *Vibrio* spp. (cells/L) measured by qPCR was found to be significantly different between site LP 1 and LP 3, LP 5, and LP 7 over the course of the study (Table S6). Additionally, detected *Vibrio* spp. for sites LP 3 and LP 4 were statistically different (Table S6).

The prevalence of *Vibrio* spp. also varied spatially in Lake Pontchartrain (Fig. 4). For example, the sampling sites LP 1 and LP 4 had consistently lower geometric means of *Vibrio* spp., while sites LP 3 and LP 8 both had geometric means greater than 2.0 × 10^4^ CFU/L (Fig. 4). The site LP 3 had the highest concentration of *Vibrio* DNA, which matched the trend for culturable *Vibrio*. Furthermore, qPCR data revealed that LP 1 had a significantly different number of *Vibrio* spp. cells compared to LP 3, LP 5, and LP 7 (Table S6).

### Correlations between the occurrence of *Vibrio* spp. and water quality

FIB, such as *E. coli* and enterococci, are commonly used as an indicator for fecal contamination in recreational water. In this study, the Pearson correlation test found only a very weak correlation (r < 0.2) between FIB and the detection of *Vibrios* in water samples. The months when average FIB concentrations were at their peak (March 2024, April 2024, and October 2024) were not the same months when *Vibrio* spp. concentrations were highest (May 2024, June 2024, and July 2024) (Table S4; Fig. 3).

The linear models between *Vibrio* concentrations and environmental factors showed that salinity and temperature were significant predictors of *Vibrio* concentrations from both bacterial culture and direct qPCR methods (Table 2). Additionally, precipitation was a significant predictor for culturable *Vibrio* spp. in this study (Fig. S2). The model for bacterial culture concentration had a higher adjusted *r*^2^ value compared to the qPCR model (0.3914 and 0.1401, respectively), meaning that concentrations from bacterial culture methods were better predicted by environmental factors. Furthermore, a Pearson correlation test shows that *Vibrio* concentrations as determined using the culture method and direct qPCR were weakly correlated with an *r*^2^ value of 0.2585.

**TABLE 2:** Linear models comparing *Vibrio* quantification and environmental parameters

	Environmental parameter	*P*-value	Model adjusted *r*^2^
*Vibrio* spp. bacterial culture concentration (CFU/L)	Salinity (psu)	0.005463^*a*^	0.3914
	Temperature (°C)	5.791 × 10^−9^^*a*^	
	7-day precipitation (in.)	0.006542^*a*^	
	Dissolved oxygen (%)	0.4570	
	Total dissolved solids (ppm)	0.5755	
	pH	0.5946	
*Vibrio* spp. qPCR concentration (cells/L)	Salinity (psu)	0.0330^*a*^	0.1401
	Temperature (°C)	0.02439^*a*^	
	7-day precipitation (in.)	0.7772	
	Dissolved oxygen (%)	0.7320	
	Total dissolved solids (ppm)	0.7727	
	pH	0.8208	

^*a*^ Indicates a statistical significance (*P*-value <0.05).

Additional temporal analysis focused on samples collected from the south shore sites (LP 4–LP 9) in September 2024, the week following Hurricane Francine, revealed that several environmental parameters were significantly different compared to samples collected at the same sites later in Fall 2024. Specifically, salinity, rainfall, and temperature differed significantly between September and the months of October and November 2024 (*P* < 0.05). However*, Vibrio* spp. concentrations, as measured by both bacterial culture and qPCR methods, were not significantly different across these time points (*P* > 0.05). In contrast, a parametric t-test showed a significant difference in culturable *Vibrio* concentrations when comparing water samples collected in November 2024 to those collected in November 2023, a period when no named storms impacted the New Orleans area. Average *Vibrio spp*. concentrations measured by culture were 5.1 × 10⁴ CFU/L in November 2023 and 1.2 × 10⁴ CFU/L in November 2024, a statistically significant difference (*P* = 0.047).

### Presence of *Vibrio* species of concern

**Fig 5:**
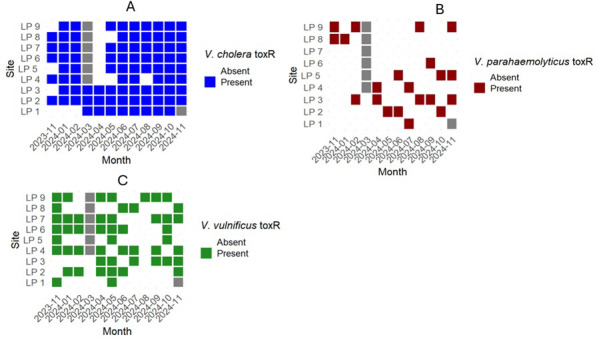
*Vibrio* spp. *toxR* genes were detected in every sampling month over the course of the study. Spatiotemporal variability of culturable (**A**) *V. cholerae*, (**B**) *V. parahaemolyticus*, and (**C**) *V*. *vulnificus* in an estuary as confirmed using conventional PCR targeting the *toxR* gene. Gray squares represent time periods when samples were not collected.

Conventional PCR targeting the *toxR* gene, a transcriptional regulator of virulence and survival genes, was used to confirm and identify culturable *Vibrio* species of concern. Among the samples collected (*n* = 101), 85.2% (*n* = 86) contained at least one colony that tested positive for *V. cholerae toxR* gene, 20.8% (*n* = 21) for *V. parahaemolyticus toxR* gene, and 50.5% (*n* = 51) for *V. vulnificus toxR* gene (Fig. 5; Table S7). Notably, nine samples (8.9%) contained at least one colony that was positive for the *toxR* gene of all three species of concern. In June, July, September, and October 2024, all sampling sites contained at least one colony positive for the *V. cholerae toxR* gene (Fig. 5).

**Fig 6:**
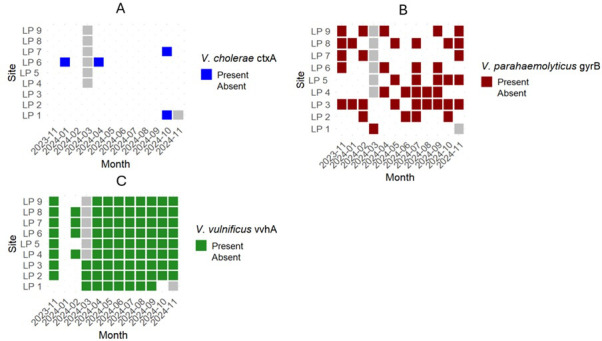
Genes essential for toxin production *V. cholerae ctxA* and *V. vulnificus vvhA* were detected in water samples from Lake Pontchartrain. Spatiotemporal variability of (**A**) *V. cholerae ctxA* gene, (**B**) *V. parahaemolyticus gyrB* gene, and (**C**) *V. vulnificus vvhA* gene in an estuary as confirmed by qPCR. Gray squares represent time periods when samples were not collected.

Quantitative PCR assays were also used for the direct detection of *Vibrio* species of concern, using DNA extracted from filters without any culture steps (Table S7). In contrast to the detection of the *toxR* gene, the *V. cholerae ctxA* gene, which codes for the A subunit of the cholera toxin, was only found in four samples (3.9%), with concentrations ranging from 33.24 to 105.8 gene copies per liter (GC/L) (Fig. 6; Table S7). The *V. parahaemolyticus gyrB* gene, which encodes this species’ DNA gyrase and appears a single time in the genome of each organism, was detected in 41.6% of samples, with concentrations ranging from 12.28 to 1.28 × 10^4^ GC/L. With direct qPCR, there was no clear trend in times of year or specific sites where *V. parahaemolyticus gyrB* gene was more prevalent (Fig. 6; Table S7).

**Fig 7:**
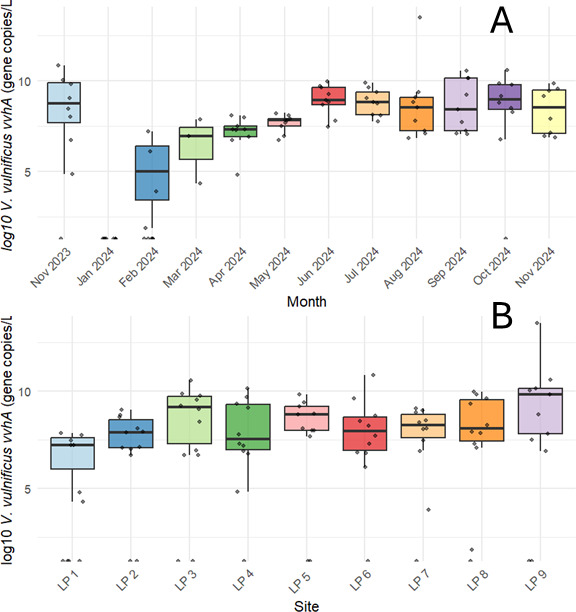
*Vibrio vulnificus* hemolysin (*vvhA*) gene showed significant temporal variation across the study months. The concentration of *V. vulnificus vvhA* gene in an estuary. Quantitative PCR showing (**A**) temporal and (**B**) spatial distributions of the gene. Each dot represents an individual sample. Box boundaries represent the lower (Q1) and upper (Q3) quartiles, and whiskers extend to the minimum and maximum non-outlier values. Pairwise comparisons showed significant differences between *vvhA* gene copies/L in winter months (January and February) and spring, summer, and fall (Table S9).

Direct qPCR revealed a high prevalence of the single-copy *V. vulnificus vvhA* gene, which encodes a hemolysin protein associated with pathogenicity. The gene was detected in 84.2% (*n* = 85) of samples, with concentrations ranging from 6.49 to 7.21 × 10⁵ GC/L (Fig. 6; Table S7). Temporal patterns were evident: no *vvhA* gene was detected in January 2024, while all samples from April through September 2024 were tested positive (Fig. 6). The highest concentrations occurred from June to September, ranging from 9.23 × 10^2^ to 7.21 × 10⁵ GC/L (Fig. 7A; Table S7). The only months that there were not significant differences in *vvhA* concentrations between January 2024 were February and March 2024 (Fig. 5 and 6; Table S9). Significant differences were also seen between February 2024 and July, August, and September 2024 (Table S9). Spatially, the highest concentrations were observed at sites LP 4, LP 6, LP 7, and LP 8—all located on the south shore of Lake Pontchartrain (Fig. 7B; Table S6).

### Diversity of *Vibrio* spp. in an estuarine lake

Four sequencing libraries were created from the 60 different samples that were barcoded for long-read nanopore sequencing. For sequencing runs that failed before 72 h, the same library was run again to generate more sequencing reads. In total, 7 runs were used in this study, with a total of 146 million reads generated and 534.86 Gb of DNA sequenced (Table S11). The N50 values for the reads ranged from 4.89 to 8.31 kb.

**Fig 8:**
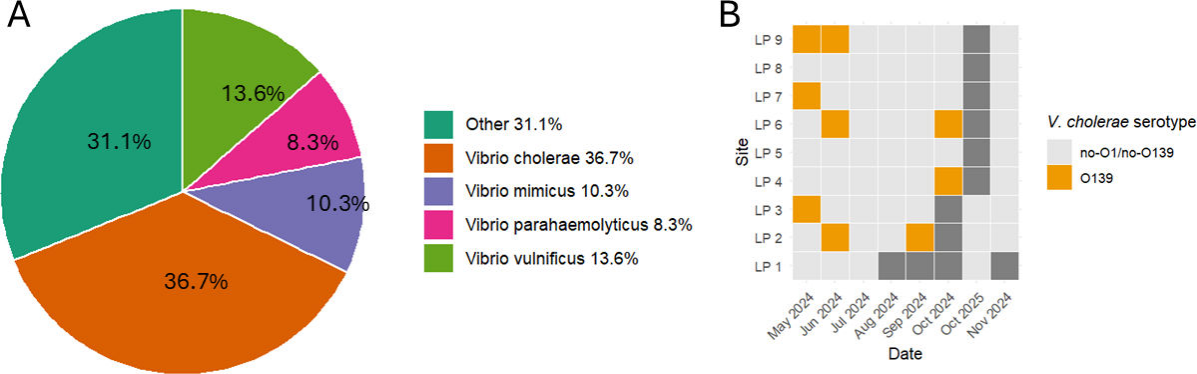
Bacterial isolate sequencing revealed that *V. cholerae*, *V. vulnificus, V. mimicus,* and *V. parahaemolyticus* made up the majority of *Vibrio* spp. communities in the estuary, with the presence of *V. cholerae* O139 serotype. (**A**) Pie chart showing the proportion of sequenced nucleotides that corresponds to *Vibrio* spp. in the samples. Species that made up <1% of total nucleotides were placed in the “Other *Vibrio* spp.” category. (**B**) Heatmap showing sampling dates and sites when *V. cholerae* O139 was detected from pooled isolate samples. Gray squares represent instances when samples were not sequenced.

Sequencing bacterial colonies pooled from 60 sample culture plates and comparing them to known sequences in the NCBI GenBank database revealed that the majority of the reads aligned with *V. cholerae* genomes (Fig. 8). Of the total nucleotides sequenced, 36.7% were identified as *V. cholerae*, followed by *V. vulnificus* with 13.6%, and *V. mimicus* with 10.3%. The “Other” category contains those species that contributed less than 1% of the sequenced nucleotides, which includes over 37 other known *Vibrio* species (Fig. 8; Table S12). Of the pooled isolate samples (*n* = 60), nine samples contained reads that aligned with the *wbf* gene cluster, indicative of the O139 serotype (Fig. 8).

## DISCUSSION

Previous reports in Louisiana have focused on the surveillance of *Vibrio* infections (38) and culturable *Vibrio* spp. in oysters from the Gulf of Mexico (39). One previous study in Lake Pontchartrain focused on *Vibrio* concentrations in offshore and canal sampling sites, following major hurricanes (26). This study instead focused on the occurrence and diversity of *Vibrio* spp. in brackish water from Lake Pontchartrain, specifically in recreational areas. At these sites, the mean culturable *Vibrio* spp. concentrations were found to be highest during the months of May, June, and July, which aligns with results from the linear model comparing these concentrations and environmental factors. The linear model showed that temperature, salinity, and precipitation were significant predictors of culturable *Vibrio* concentration. This is in line with other studies of environmental *Vibrio* that have noted a linear relationship between temperature and *Vibrio* concentrations (9, 40, 41).

Similarly, a linear model showed that both temperature and salinity were significant predictors of *Vibrio* 16S rRNA gene concentrations. However, the *r*^2^ values for these models show that all environmental factors included in the model explained about 39% of the culturable *Vibrio* spp. variation and only about 14% of the qPCR *Vibrio* concentration. It is possible that gene concentrations would be less correlated with environmental factors, as *Vibrio* spp. can exist in a dormant, nonculturable state when environmental conditions fluctuate and can still be detected by qPCR (19). *Vibrio* spp. can survive at temperatures as low as 4°C by entering this dormant state, but optimum growth occurs at temperatures between 25°C and 30°C. It is possible that this VBNC state contributed to the lower *r*^2^ value for the qPCR model, as this method would account for bacteria in a metabolically dormant state during the winter months (10). This is further evidenced by the significant differences in *Vibrio* spp. abundance between January and February 2024 and May, June, and July 2024 for cultured colonies, but not concentrations as determined by qPCR, showing that fluctuations in culturable *Vibrio* spp. concentrations do not directly reflect changes in the total amount of *Vibrio* genes. Even with all six environmental factors included in the model (salinity, temperature, precipitation, DO, TDSs, and pH), less than half of the fluctuation in culturable *Vibrio* spp. concentrations was explained. These results show the importance of monitoring *Vibrio* spp. in recreational waters throughout the year, as environmental factors alone are insufficient for predicting potential risk of exposure to recreators.

The Louisiana Department of Health (LDH) monitors 23 beaches across the state, including one site on the northern Lake Pontchartrain, for enterococci as indicators for fecal contamination, while the Louisiana Department of Environmental Quality (DEQ) continuously monitors a site in the center of the lake for fecal coliforms (Louisiana BEACH Grant Report 2024 Swimming Season, 2024; Louisiana Water Quality Inventory: Integrated Report, 2024). The LDH and DEQ use these measures to make advisories about beach safety, but neither entity conducts any water testing for *Vibrio* in Louisiana beaches because *Vibrio* are naturally present in warm coastal water. In this study, many recreational sites were tested for FIB and held to the EPA recommendation of less than 35 *Enterococci* per 100 mL and less than 126 *E. coli* per 100 mL of water sample. Even when FIB levels were below these limits, pathogenic *Vibrio* spp. were detected in water samples. For water samples that exceeded the EPA standard levels for FIB, there was a very weak correlation between *Vibrio* concentrations and FIB. Studies on the correlation between FIB and *Vibrio* abundance have shown mixed results, with some showing increased counts of *Vibrio* near sources of fecal contamination and others showing no correlation between the two types of bacteria (42, 43). This again underscores the need for *Vibrio* testing, as environmental factors or FIB presence cannot fully predict the variability of these bacteria in coastal recreational waters.

In addition to normal seasonal variations in environmental parameters, Hurricane Francine impacted the city of New Orleans during September 2024. This event caused significant differences between salinity, rainfall, and temperature for this month compared to other samples collected during the Fall of 2024. A comparison between November 2024, after this extreme weather event, and the previous November, when New Orleans experienced no named tropical disturbances, shows that the *Vibrio* spp. was significantly lower in November 2024. This differs from a study conducted after Hurricane Ian made landfall in Florida in 2022, which showed that environmental changes due to the storm made the waters more favorable for *Vibrio* spp. growth (44). However, it is not possible to compare *Vibrio* spp. concentrations from September 2023 to determine whether *Vibrio* spp. concentrations may have been higher immediately following Hurricane Francine. While in this study, comparison could only be made 2 months after the hurricane, it is important to continue to study how these extreme weather events can influence *Vibrio* spp. populations.

For the samples collected in this study, *V. cholerae toxR* gene was present in most samples, followed by *V. vulnificus toxR* gene, and finally *V. parahaemolyticus toxR* gene. This is corroborated by past research on water samples from Lake Pontchartrain and the Gulf of Mexico, where these three species of *Vibrio* were present (25, 26). However, epidemiological data suggest that *V. parahaemolyticus* is the most commonly reported cause of vibriosis in Louisiana and the U.S. (7, 22, 45), but *V. parahaemolyticus toxR* gene was only present in 14% of samples. This could be attributed to the fact that *V. parahaemolyticus* tends to be more associated with shellfish, particularly oysters, while *V. cholerae* is found more often in the water column (16). While *V. parahaemolyticus* was found in fewer water samples, its association with seafood could explain the higher rates of infection.

Quantitative PCR results for specific toxin genes of *V. cholerae* and *V. vulnificus*, cholera toxin, and *V. vulnificus* hemolysin, respectively, show that these are not directly correlated to the presence of the *toxR* gene in either of these species. In this study, the cholera toxin gene (*ctxA*), which encodes the cholera toxin responsible for potentially deadly diarrhea and epidemics around the world (46, 47), was only found in 4% of samples. However, in 128 samples collected in Lake Pontchartrain between 2005 and 2006, none tested positive for the *ctxA* gene (26). This could be due to changing conditions in the lake or because, in this study, the *ctxA* gene was identified using qPCR, which can identify the gene even if the bacteria are in a VBNC state. The *ctxA* gene is essential for expression of the cholera toxin protein, and this gene has been associated with all forms of pandemic *V. cholerae* (47). Although cholera incidence in the U.S. has remained low in recent decades, environmental shifts that have the potential to affect the ecology of *Vibrio cholerae* underscore the need for continued monitoring of this pathogen in coastal waters (22, 48).

While both the *V. vulnificus* hemolysin gene and the *Vibrio* 16S rRNA concentrations peaked during summer months, they did not peak at the same time. This could mean that the risk of interaction with toxigenic *Vibrio* spp. persists in months when total *Vibrio* populations are lower. Even in times when there are lower concentrations of *Vibrio* bacteria in the water, recreators still interface with toxin-producing species. Additionally, the site with the highest concentration of both culturable *Vibrio* spp. and *Vibrio* 16S rRNA genes, LP 3, did not contain the highest concentration of *V. vulnificus* hemolysin genes. This shows that the spatial distribution of pathogenic *Vibrio* could differ from the overall distribution of *Vibrio* species. Although long-read nanopore sequencing has recently been used to study organismal isolates, this is the first time it has been implemented as a continuous surveillance tool across recreational water sites (49, 50). Analysis of sequences revealed that the *V. cholerae* made up the largest proportion of culturable *Vibrio* spp. in collected samples, and the O139 serotype was present at sampling sites. This strain emerged in the 1990s and has been responsible for global epidemic outbreaks of *V. cholerae* (51). Cholera infections make up a very small proportion of total reported *Vibrio* infections in the U.S (22), and there has only been one confirmed *V. cholerae* O139 case in Louisiana since 1988 (45). Furthermore, sequencing of the pooled *Vibrio* colonies revealed 41 species of *Vibrio,* including *V. mimicus*, which is very similar to *V. cholerae* and can contain the genes for the cholera toxin (52, 53). While much of the work on *Vibrio* spp. focuses on the three species of concern, *V. cholerae, V. vulnificus, and V. parahaemolyticus*, there is a large diversity of bacteria in this genus, and it could be important to look at the dynamics between different species beyond those most commonly studied. Identifying other *Vibrios* that infect humans, including *V. fluvialis* and *V. mimicus*, which were both detected by bacterial isolate sequencing, can be important in assessing the risk of vibriosis in recreational waters (1). Moreover, analysis of these complex bacterial populations could reveal relationships between different *Vibrio* species that could be used to further understand the spatiotemporal dynamics of pathogenic species.

One advantage of bacterial isolate genome sequencing is that it does not require primer design like target-specific PCR. This allows for the detection of all *Vibrio* spp. and serotypes in a sample, not just the pre-defined species of concern (35). Furthermore, the use of nanopore long-read sequencing allows for the identification of specific virulence genes, like the *wbz* gene specific to the *V*. cholerae O139 serotype, that can help in understanding the *Vibrio* spp. community beyond the species level. However, this method is relatively costly and time-consuming compared to more rapid detection with PCR methods. Although culture methods have long been considered the gold standard for pathogen detection, they require incubation times between 24 and 48 h, which can hinder rapid detection of *Vibrio* spp. in recreation waters. Since qPCR can be run without growing the bacteria, it can get faster results, but it tests for the presence of DNA rather than viable cells (54). This means that the qPCR technique can quantify all living *Vibrio* in a sample, even if they are metabolically dormant, but it also quantifies genetic material present in dead cells. These approaches all have their advantages and disadvantages, but they can be used together to get a comprehensive understanding of microbial communities in recreational water (54). For example, continuous monitoring of *Vibrio* concentrations and the presence of infectious *Vibrio* using a combination of culture- and molecular-based methods would facilitate more accurate assessment of the exposure risk posed to recreators in coastal water environments.

### Conclusion

*Vibrio* spp. concentrations were found to be highest in recreational water of Lake Pontchartrain in the summer, with a moderate correlation between water temperature and culturable *Vibrio* spp., but the bacteria persisted throughout the year. Furthermore, PCR and genome sequencing revealed the presence of *V. cholerae,* including the O139 serotype, *V. vulnificus*, *V. parahaemolyticus*, *V. mimicus,* and 37 of other species in these samples. It would be valuable for future studies to determine other environmental parameters that influence the variation in *Vibrio* spp. populations and the interactions between the different bacterial species that make up the *Vibrio* community in these waters. This study has broader public health and environmental implications as future climate scenarios suggest that summers will become longer and warmer, increasing the exposure risk of pathogenic *Vibrio* during recreational activities (15, 40, 48).

## Data Availability

The sequencing data of this study have been deposited in the NCBI database under BioProject PRJNA1414565 (https://www.ncbi.nlm.nih.gov/sra/PRJNA1414565).

## SUPPLEMENTAL MATERIAL

Supplemental material aem.01944-25-s0001.docx Tables S1 to S12; Fig. S1 to S3.

Graphical abstract aem.01944-25-s0002.tiff Visual depiction of the study.

ASM does not own the copyrights to Supplemental Material that may be linked to, or accessed through, an article. The authors have granted ASM a non-exclusive, world-wide license to publish the Supplemental Material files. Please contact the corresponding author directly for reuse.
