# Supplementary material for: Spatiotemporal distribution and diversity of pathogenic Vibrio species in estuarine recreational waters of southeast Louisiana
Source: Appl Environ Microbiol. 2026 Feb 11;92(3):e01944-25. doi: 10.1128/aem.01944-25 (PMC12997861; doi:10.1128/aem.01944-25)
Supplement: Supplemental material — Tables S1 to S12; Fig. S1 to S3. [file aem.01944-25-s0001.docx]

**Supplementary Materials**

**Spatiotemporal distribution and species diversity of pathogenic *Vibrios* in estuarine recreational waters of southeast Louisiana**

Annika Nelson, Fernanda Mac-Allister Cedraz, Katie Vigil, Joshua Alarcon, Tiong Gim Aw^*^

Department of Environmental Health Sciences, Celia Scott Weatherhead School of Public Health and Tropical Medicine, Tulane University, New Orleans, Louisiana, USA

^*^Corresponding author:

Tiong Gim Aw

Mailing address:

Department of Environmental Health Sciences

School of Public Health and Tropical Medicine

Tulane University
1440 Canal Street, Suite 2100
New Orleans, LA 70112, USA

Phone: +1 504-988-9926

E-mail: [taw@tulane.edu](mailto:taw@tulane.edu)

**Table S1.** Sampling location coordinates

| **Site** | **Shore** | **Latitude** | **Longitude** |
| --- | --- | --- | --- |
| **LP 1** | North | 30°22'42.52"N | 90° 9'38.37"W |
| **LP 2** | North | 30°20'5.75"N | 90° 2'42.49"W |
| **LP 3** | North | 30°13'27.20"N | 89°50'8.60"W |
| **LP 4** | South | 30° 6'25.68"N | 90°25'21.62"W |
| **LP 5** | South | 30° 2'27.49"N | 90°14'18.01"W |
| **LP 6** | South | 30° 1'14.81"N | 90° 8'31.65"W |
| **LP 7** | South | 30° 1'42.69"N | 90° 6'45.59"W |
| **LP 8** | South | 30° 1'53.47"N | 90° 2'16.94"W |
| **LP 9** | South | 30° 8'28.39"N | 89°51'47.53"W |


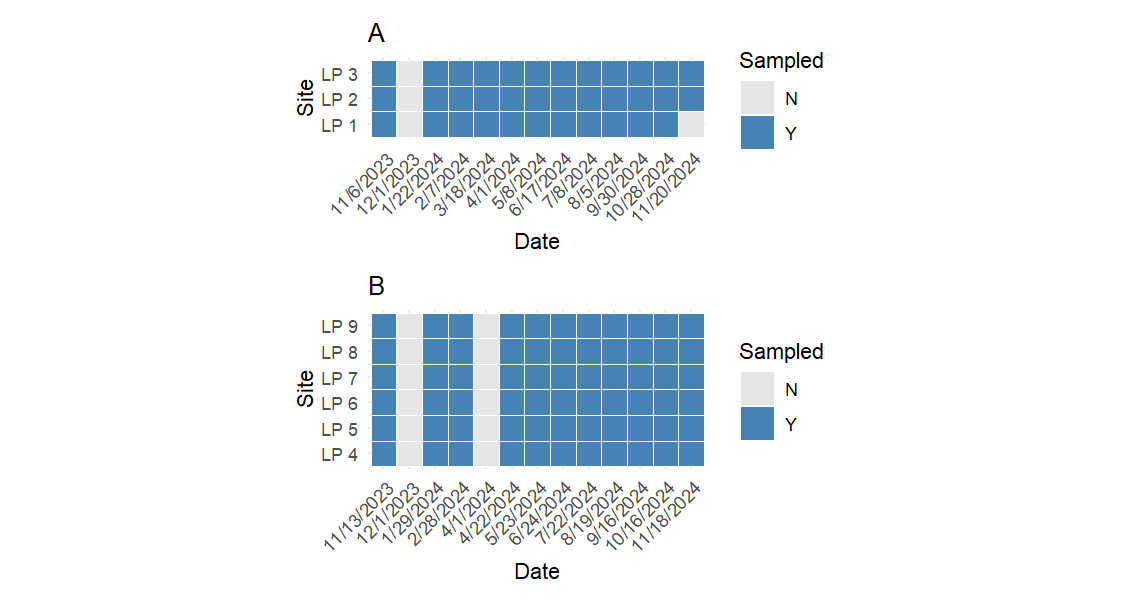


**Figure S1.** Heat map showing dates when samples were collected from each sampling site. Two sampling trips were conducted each month of the study, one for the North Shore sites (A) and one for the South Shore sites (B). Dates with sample collected are signified in blue (Y) and sample not collected are signified in grey (N).

**Table S2**. Primers, probes, and gBlocks standards for *Vibrio* PCR and qPCR assays.

| **Species** | **Target Gene** | **Assay** | **Component** | **DNA Sequence (5'-3')** | **Reference** |
| --- | --- | --- | --- | --- | --- |
| ***Vibrio* genus** | 16S rRNA | SYBR Green qPCR | F | GGCGTAAAGCGCATGCAGGT | (Thompson et al., 2004) |
|  |  |  | R | GAAATTCTACCCCCCTCTACAG | (Thompson et al., 2004) |
|  |  |  | S | CTGACGAGCGGCGGACGGGTGAGTAATGCCTGGGAATATGCCCTGATGTGGGGGATAACTATTGGAAACGATAGCTAATACCGCATAATCTCTTCGGAGCAAAGAGGGGGACCTTCGGGCCTCTCGCGTCAGGATTAGCCCAGGTGGGATTAGCTTGTTGGTGGGGTAATGGCTCACCAAGGCAACGATCCCTAGCTGGTCTGAGAGGATGATCAGCCACACTGGAACTGAGACACGGTCCAGACTCCTACGGGAGGCAGCAGTGGGGAATATTGCACAATGGGGGAAACCCTGATGCAGCCATGCCGCGTGTATGAAGAAGGCCTTCGGGTTGTAAAGTACTTTCAGTTGTGAGGAAGGCGTTGATGTTAATAGCTTCAGCGTTTGACGTTAGCAACAGAAGAAGCACCGGCTAACTCCGTGCCAGCAGCCGCGGTAATACGGAGGGTGCGAGCGTTAATCGGAATTACTGGGCGTAAAGCGCATGCAGGTGGTCTGTTAAGCAAGATGTGAAAGCCCGGGGCTCAACCTCGGAACAGCATTTTGAACTGGCAGACTAGAGTACTGTAGAGGGGGGTAGAATTTCAGGTGTAGCGGTGAAATGCGTAGAGATCTGAAGGAATACCGGTGGCGAAGGCGGCCCCCTGGACAGATACTGACACTCAGATGCGAAAGCGTGGGGAGCAAACAGGATTAGATACCCTGGTAGTCCACGCCGTAAACGATGTCTACTTGGAGGTTGTGGCCTTGAGCCGTGGCTTTCGGAGCTAACGCGTTAAGTAGACCGCCTGGGGAGTACGGTCGCAAGATTAAAACTCAAATGAATTGACGGGGGCCAGAAAGTACGTCGTAGTCCGGATTGGAGTCTGCAACTCGACTCCATGAAGTCGGAATCGCTAGTAATCGTGAATCAGAATGTCACGGTGAATACGTTCCCGGGCCTT |  |
| ***Vibrio* genus** | toxR | PCR | F | GASTTTGTTTGGCGYGARCAAGGTT | (Bauer & Rørvik, 2007) |
| ***V. parahaemolyticus*** | toxR | PCR | R | GGTTCAACGATTGCGTCAGAAG | (Bauer & Rørvik, 2007) |
| ***V. cholerae*** | toxR | PCR | R | GGTTAGCAACGATGCGTAAG | (Bauer & Rørvik, 2007) |
| ***V. vulnificus*** | toxR | PCR | R | AACGGAACTTAGACTCCGAC | (Bauer & Rørvik, 2007) |
| ***V. cholerae*** | *ctxA* | qPCR | F | TTTGTTAGGCACGATGATGGAT | (Blackstone et al., 2007) |
|  |  |  | R | ACCAGACAATATAGTTTGACCCACTAAG | (Blackstone et al., 2007) |
|  |  |  | P | FAM-TGTTTCCACCTCAATTAGTTTGAGAAGTGCCC-BHQ1 | (Blackstone et al., 2007) |
|  |  |  | S | GTCAGGTGGTCTTATGCCAAGAGGACAGAGTGAGTACTTTGACCGAGGTACTCAAATGAATATCAACCTTTATGATCATGCAAGAGGAACTCAGACGGGATTTGTTAGGCACGATGATGGATATGTTTCCACCTCAATTAGTTTGAGAAGTGCCCACTTAGTGGGTCAAACTATATTGTCTGGTCATTCTACTTATTATATATATGTTATAGCCACTGCACCCAACATGTTTAACGTTAATGATGTATTAGGGGCATACAGTCCTCATCCAGATGAACAAGAAG |  |
| ***V. vulnificus*** | *vvhA* | qPCR | F | TGCCTRGATGTTTATGGTGAGAAC | (Campbell & Wright, 2003) |
|  |  |  | R | TCGACTGTGAGCGTTTTGTC | (Campbell & Wright, 2003) |
|  |  |  | P | FAM-TAGCCGAGTRGCATCCGATCGTTGTT-BHQ1 | (Campbell & Wright, 2003) |
|  |  |  | S | ACTATCGTGCACGCTTTGGTACCGTTCTTCCTTCAGCGCTGTTTTCGGTTTACGGCTCTGCGGGCTCGTCAACCAACAGCAGTACTGTGAAACAACGTATTCGCATCGACTGGAATCACCCACTGTTTGAAGCGGAAGCACACGTTACACTACAGTCACTGAGCAACAACGATCTCTGCCTAGATGTTTATGGTGAGAACGGTGACAAAACGGTTGCGGGTGGTTCGGTTAACGGCTGGAGCTGTCACGGCAGTTGGAACCAAGTTTGGGGCCTAGATAAAGAAGAACGTTATCGTAGCCGAGTGGCATCCGATCGTTGTTTGACCGTAAACGCTGACAAAACGCTCACAGTCGAACAGTGTGGTGCGAACTTAGCACAGAAATGGTATTGGGAAGGCGATAAGCTCATTAGCCGCTATGTTGATGGCAGT |  |
| ***V. parahaemolyticus*** | *gyrB* | qPCR | F | TGAAGGTTTGACTGCCGTTGT | (Cai et al., 2006) |
|  |  |  | R | TGGGTTTTCGACCAAGAACTCA | (Cai et al., 2006) |
|  |  |  | P | FAM-TTCTCACCCATCGCCGATTCAACCGC-BHQ1 | (Cai et al., 2006) |
|  |  |  | S | GGCAATGCAGTGGAACGATGGTTTCCAAGAGAACATCTTCTGTTTCACCAACAACATTCCACAGCGCGATGGTGGTACTCACCTTGCTGGTTTCCGTGCGGCACTAACACGTACGCTAAACAGCTTTATGGATAAAGAAGGCTTCTCGAAGAAAGCGAAAACAGCAACGTCAGGCGATGATGCGCGTGAAGGTTTGACAGCCGTTGTTTCGGTAAAAGTGCCTGATCCAAAATTCTCGAGCCAAACCAAAGACAAACTGGTTTCTTCTGAAGTGAAATCAGCGGTTGAATCGGCGATGGGTGAGAAATTATCTGAGTTCTTGGTCGAAAACCCAAGCGAAGCGAAGATGGTTTGTTCGAAAATCATCGATGCAGCACGTGCACGTGAAGCCGCACGTAAAGCGCGTGAAATGACTCGTCGTAAAGGCGCGCTAGACCTAGCAGGCCTACCAGGCAAACTTGCAGACTGTCAGGAAAAAGATCCGG |  |

F= forward primer, R= reverse primer, P=probe, and S= gBlocks DNA standard

**Table S3.** PCR and qPCR cycling conditions.

| **Assay** | **Cycle component** | **Temperature (**°C) | **Time** | **Number of Cycles** |
| --- | --- | --- | --- | --- |
| *Vibrio cholerae* PCR for *toxR* gene | Initial denaturation | 94 | 90 s | 1x |
|  | Denaturation | 94 | 30 s | 40x |
|  | Annealing | 58 | 60 s |  |
|  | Extension | 72 | 30 s |  |
| *Vibrio vulnificus* PCR for *toxR* gene | Initial denaturation | 94 | 90 s | 1x |
|  | Denaturation | 94 | 30 s | 40x |
|  | Annealing | 58 | 60 s |  |
|  | Extension | 72 | 39 s |  |
| *Vibrio parahaemolyticus* PCR for *toxR* gene | Initial denaturation | 94 | 90 s | 1x |
|  | Denaturation | 94 | 30 s | 40x |
|  | Annealing | 58 | 60 s |  |
|  | Extension | 72 | 18 s |  |
| *Vibrio* spp. qPCR for 16S rRNA | Initial hold | 50 | 10 min | 1x |
|  | Denaturation | 95 | 10 s | 40x |
|  | Annealing | 55 | 20 s |  |
|  | Melt curve | Increase from 60-95 °C in 0.1 °C increments | | 1 x |
| *Vibrio cholerae* qPCR for *ctxA* gene | Initial denaturation | 94 | 120 s | 1x |
|  | Denaturation | 94 | 10 s | 40x |
|  | Annealing | 63 | 30 s |  |
| *Vibrio vulnificus* qPCR for *vvhA* gene | Initial denaturation | 95 | 120 s | 1 x |
|  | Denaturation | 95 | 15 s | 40 x |
|  | Annealing | 60 | 30 s |  |
| *Vibrio parahaemolyticus* qPCR for *gyrB* gene | Initial denaturation | 95 | 10 min | 1 x |
|  | Denaturation | 95 | 3 s | 40 x |
|  | Annealing | 60 | 20 s |  |
|  | Extension | 72 | 10 s |  |


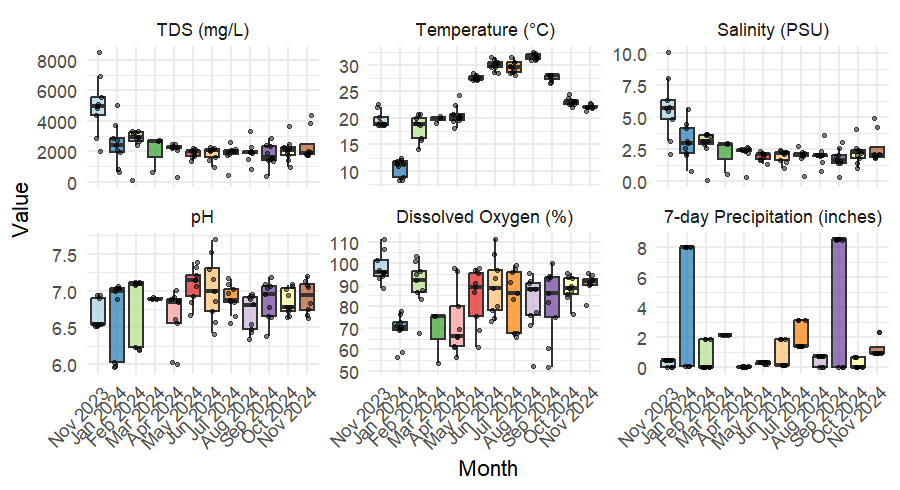


**Figure S2.** Temporal distribution of environmental parameters including TDS, temperature, salinity, pH, dissolved oxygen, and precipitation. The x-axis denotes sampling months from November 2023 to November 2024 and the y-axis denotes the measurement value for each parameter. Each dot represents an individual sample. Box boundaries represent the lower (Q1) and upper (Q3) quartiles, and whiskers extend to the minimum and maximum non-outlier values.

**Table S4.** Geometric means of fecal indicator bacteria and *Vibrio* bacteria concentrations over months in study period. All means were calculated based on 9 sampling points within that month unless otherwise indicated.

| Month | Geometric Mean of *E. coli* concentration (MPN/100 mL) | Geometric Mean of *Enterococci* concentration (MPN/100 mL) | Geometric Mean of Bacterial Culture *Vibrio* spp. concentration (CFU/100 mL) | Geometric Mean of qPCR *Vibrio* spp. concentration (cells/100 mL) |
| --- | --- | --- | --- | --- |
| November 2023 | 77.4 | 61.4 | 1540 | 2605 |
| January 2024 | 204* | 90.9 | 126 | 769 |
| February 2024 | 37 | 67.3 | 186 | 706 |
| March 2024 | 10.6* | 478* | 613* | 1463* |
| April 2024 | 17.4 | 112 | 1760 | 1804 |
| May 2024 | 28.5 | 59.2 | 6220 | 4392 |
| June 2024 | 18.1 | 86.4 | 5180 | 2192 |
| July 2024 | 30.9 | 59.2 | 4410 | 2638 |
| August 2024 | 10.9 | 51.4 | 1570 | 1466 |
| September 2024 | 20.3 | -*** | 508 | 1180 |
| October 2024 | 28.3 | 124 | 2840 | 2561 |
| November 2024 | 105** | 45.1** | 1217** | 5082** |

* Only 3 sites tested in this month for this parameter.

**Only 8 sites tested in this month for this parameter.

***No samples tested in this month for this parameter.

**
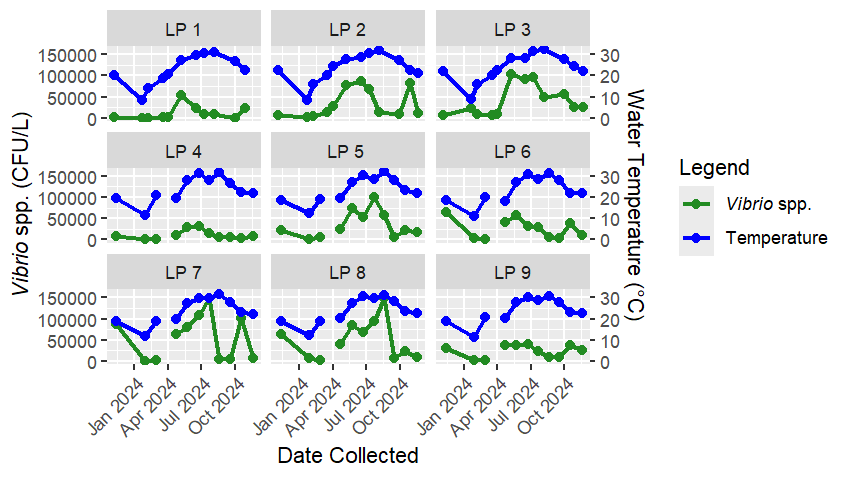
**

**Figure S3.** Correlations of water temperature and *Vibrio* spp. concentration over time. Enumeration from culture methods over time (green) compared to water temperature (blue) measured in situ at each site during sample collection. The left-axis shows values for *Vibrio* spp. in CFU/L. The right-axis shows water temperature in degrees Celsius. The x-axis shows condensed sampling timeline, and each dot is a sampling event.

**Table S5.** Paired months showing significant differences in *Vibrio* spp. concentrations measured by bacterial culture methods in CFU per liter.

| Months Compared | Wilcox adjusted p-value |
| --- | --- |
| Jan 2024:May 2024 | 0.003** |
| Jan 2024:Jun 2024 | 0.003** |
| Jan 2024:Jul 2024 | 0.019* |
| Jan 2024:Oct 2024 | 0.019* |
| Feb 2024:May 2024 | 0.003** |
| Feb 2024:Jun 2024 | 0.003** |
| Feb 2024:Jul 2024 | 0.005** |
| Feb 2024:Oct 2024 | 0.011* |
| Feb 2024:Nov 2024 | 0.038* |
| May 2024:Sep 2024 | 0.019* |
| May 2024:Nov 2024 | 0.005** |
| Jun 2024:Nov 2024 | 0.022* |

* Denotes a p-value below 0.05.

** Denotes a p-value below 0.01.

**Table S6.** Paired months showing significant differences in *Vibrio* spp. concentrations measured by quantitative PCR in cells per liter.

| **Comparison Pair** | **Wilcox adjusted p-value** |
| --- | --- |
| LP 1:LP 3 | 0.002** |
| LP 1:LP 5 | 0.028* |
| LP 1:LP 7 | 0.014* |
| LP 3:LP 4 | 0.02* |

* Denotes a p-value below 0.05.

** Denotes a p-value below 0.01.

**Table S7.** *Vibrio* species of concern detection rates with conventional PCR and qPCR

| **Organism** | **Gene** | **Assay** | **Detection rate (%)** | **Concentration range (gene copies/L)** |
| --- | --- | --- | --- | --- |
| *V. cholerae* | ctxA | qPCR | 3.90 | 33.24 to 105.75 |
|  | toxR | PCR | 85.20 |  |
| *V. parahaemolyticus* | gyrB | qPCR | 41.60 | 12.28 to 1.28 x 10^4^ |
|  | toxR | PCR | 20.80 |  |
| *V. vulnificus* | vvhA | qPCR | 84.20 | 6.49 to 7.21 x 10^5^ |
|  | toxR | PCR | 50.50 |  |

**TABLE S8.** Temporal distribution of *Vibrio vulnificus* *vvhA* gene concentrations

| **Month** | **Geometric Mean of *vvhA* concentration (gene copies/L)** |
| --- | --- |
| January | 0 |
| February | 0 |
| March | 594.41 |
| April | 1211.86 |
| May | 2072.18 |
| June | 7471.02 |
| July | 6816.07 |
| August | 5970.54 |
| September | 5526.83 |
| October | 222.41 |
| November | 710.74 |

**Table S9.** Paired months showing significant differences in *V. vulnificus vvhA* concentrations measured by quantitative PCR in gene copies per liter.

| **Comparison Pair** | **Wilcox adjusted p-value** |
| --- | --- |
| Nov 2023:Jan 2024 | 0.038* |
| Jan 2024:Apr 2024 | 0.011* |
| Jan 2024:May 2024 | 0.011* |
| Jan 2024:Jun 2024 | 0.011* |
| Jan 2024:Jul 2024 | 0.011* |
| Jan 2024:Aug 2024 | 0.011* |
| Jan 2024:Sep 2024 | 0.011* |
| Jan 2024:Oct 2024 | 0.038* |
| Jan 2024:Nov 2024 | 0.014* |
| Feb 2024:May 2024 | 0.046* |
| Feb 2024:Jun 2024 | 0.024* |
| Feb 2024:Jul 2024 | 0.024* |
| Feb 2024:Aug 2024 | 0.046* |
| Feb 2024:Sep 2024 | 0.046* |
| Apr 2024:Jul 2024 | 0.011* |

* Denotes a p-value below 0.05.

**TABLE S10.** Spatial distribution of *Vibrio vulnificus* vvhA gene concentrations

| **Site** | **Geometric Mean of *vvhA* concentration (gene copies/L)** |
| --- | --- |
| LP 1 | 0.02 |
| LP 2 | 14.82 |
| LP 3 | 30.27 |
| LP 4 | 164.96 |
| LP 5 | 18.12 |
| LP 6 | 190 |
| LP 7 | 155.22 |
| LP 8 | 163.83 |
| LP 9 | 35.01 |

**TABLE S11.** Sequencing run results and quality parameters

| **Sequencing library** | **Number of Samples** | **Number of Reads** | **Number of Bases (Gb)** | **N50 (kb)** | **Run Time** |
| --- | --- | --- | --- | --- | --- |
| 1 | 3 | 28.77 million | 102.23 | 4.89 | 72 hrs |
| 2 | 14 | 5.93 million | 13.66 | 5.6 | 31 hrs |
| 3 | 22 | 46.7 million | 151.08 | 6.27 | 66 hrs |
| 4 | 21 | 4.42 million | 25.34 | 7.85 | 9 hrs |
| 2 | 14* | 54.89 million | 153.72 | 5.93 | 72 hrs |
| 3 | 22* | 4.64 million | 13.98 | 6.08 | 72 hrs |
| 4 | 21* | 602,850 | 3.56 | 8.31 | 72 hrs |
| **Total** | **60*** | **146 million** | **534.86** |  |  |

*Samples from the same sequencing library were the same extraction resequenced to get a higher number of reads.

**TABLE S12.** *Vibrio* species detected by a long-read sequencing. All *Vibrio* species detected by long-read sequencing and matched with known nucleotide sequences in the NCBI GenBank nucleotide and protein database and with more than 1 kb sequenced and >95% identity. .

| **Species** | **Nucleotides Sequenced** | **Percent of Total Nucleotides Sequenced**  **(%)** | **Percent Identification (%)** |
| --- | --- | --- | --- |
| *Vibrio cholerae* | 1.14E+10 | 36.67938 | 95.4 |
| Other *Vibrio* spp. | 9.65E+09 | 30.81012 | 95.64 |
| *Vibrio vulnificus* | 4.24E+09 | 13.58555 | 95.29 |
| *Vibrio mimicus* | 3.21E+09 | 10.28862 | 95.4 |
| *Vibrio parahaemolyticus* | 2.6E+09 | 8.34294 | 95.84 |
| *Vibrio fluvialis* | 19893603 | 0.115113 | 95.37 |
| *Vibrio paracholerae* | 17569142 | 0.06374 | 95.29 |
| *Vibrio cidicii* | 6571456 | 0.056293 | 95.11 |
| *Vibrio antiquarius* | 3026306 | 0.021055 | 95.35 |
| *Vibrio diabolicus* | 2151836 | 0.009696 | 95.03 |
| *Vibrio nigripulchritudo* | 1830087 | 0.006895 | 95.54 |
| *Vibrio neocaledonicus* | 1154440 | 0.005864 | 95.92 |
| *Vibrio cincinnatiensis* | 906632 | 0.003699 | 95.26 |
| *Vibrio harveyi* | 667957 | 0.002905 | 95.3 |
| *Vibrio rotiferianus* | 642432 | 0.00214 | 96.17 |
| *Vibrio phage* | 274623 | 0.002058 | 95.95 |
| *Vibrio gazogenes* | 250686 | 0.00088 | 95.78 |
| *Vibrio porteresiae* | 169351 | 0.000803 | 95.93 |
| *Vibrio pectenicida* | 80318 | 0.000543 | 95.48 |
| *Vibrio ponticus* | 79520 | 0.000257 | 96.65 |
| *Vibrio casei* | 63641 | 0.000255 | 95.46 |
| *Vibrio syngnathi* | 58960 | 0.000204 | 95.34 |
| *Vibrio pomeroyi* | 56886 | 0.000189 | 95.72 |
| *Vibrio ostreae* | 43733 | 0.000182 | 95.48 |
| *Vibrio scophthalmi* | 34070 | 0.00014 | 96.58 |
| *Vibrio gallicus* | 20706 | 0.000109 | 96.75 |
| *Vibrio gigantis* | 16205 | 6.63E-05 | 95.66 |
| *Vibrio ziniensis* | 14628 | 5.19E-05 | 95.79 |
| *Vibrio tritonius* | 12242 | 4.69E-05 | 98.08 |
| *Vibrio rumoiensis* | 8876 | 3.92E-05 | 95.46 |
| *Vibrio cortegadensis* | 8506 | 2.84E-05 | 96.01 |
| *Vibrio crassostreae* | 6582 | 2.73E-05 | 96.08 |
| *Vibrio azureus* | 6162 | 2.11E-05 | 95.28 |
| *Vibrio tarriae* | 5868 | 1.97E-05 | 95.21 |
| *Vibrio kanaloae* | 5865 | 1.88E-05 | 95.11 |
| *Vibrio pelagius* | 4695 | 1.88E-05 | 98.36 |
| *Vibrio gangliei* | 2300 | 1.5E-05 | 95.69 |
| *Vibrio neptunius* | 1560 | 7.37E-06 | 96.2 |
| *Vibrio tubiashii* | 1225 | 5E-06 | 97.22 |
| *Vibrio artabrorum* | 1150 | 3.92E-06 | 95.91 |

**References**

Bauer, A., & Rørvik, L. M. (2007). A novel multiplex PCR for the identification of Vibrio

parahaemolyticus, Vibrio cholerae and Vibrio vulnificus. *Lett Appl Microbiol*, *45*(4), 371-375. <https://doi.org/10.1111/j.1472-765X.2007.02195.x>

Blackstone, G. M., Nordstrom, J. L., Bowen, M. D., Meyer, R. F., Imbro, P., & DePaola, A. (2007). Use of a real time PCR assay for detection of the ctxA gene of Vibrio cholerae in an environmental survey of Mobile Bay. *J Microbiol Methods*, *68*(2), 254-259. <https://doi.org/10.1016/j.mimet.2006.08.006>

Cai, T., Jiang, L., Yang, C., & Huang, K. (2006). Application of real-time PCR for quantitative detection of Vibrio parahaemolyticus from seafood in eastern China. *FEMS Immunol Med Microbiol*, *46*(2), 180-186. <https://doi.org/10.1111/j.1574-695X.2005.00016.x>

Campbell, M. S., & Wright, A. C. (2003). Real-time PCR analysis of Vibrio vulnificus from oysters. *Appl Environ Microbiol*, *69*(12), 7137-7144. <https://doi.org/10.1128/aem.69.12.7137-7144.2003>

Thompson, J. R., Randa, M. A., Marcelino, L. A., Tomita-Mitchell, A., Lim, E., & Polz, M. F. (2004). Diversity and dynamics of a north atlantic coastal Vibrio community. *Appl Environ Microbiol*, *70*(7), 4103-4110. <https://doi.org/10.1128/aem.70.7.4103-4110.2004>
