# Supplementary figures and images for: Spatiotemporal distribution and diversity of pathogenic Vibrio species in estuarine recreational waters of southeast Louisiana
Source: Appl Environ Microbiol. 2026 Feb 11;92(3):e01944-25. doi: 10.1128/aem.01944-25 (PMC12997861; doi:10.1128/aem.01944-25)

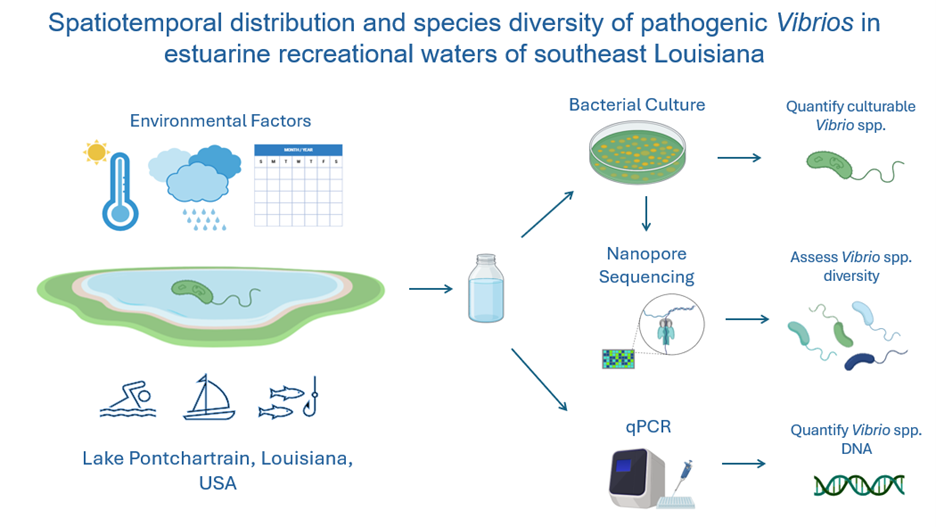

Supplement: Graphical abstract — Visual depiction of the study. [file aem.01944-25-s0002.tiff]
